# Infection prevalence and ecotypes of *Anaplasma phagocytophilum* in moose *Alces alces*, red deer *Cervus elaphus*, roe deer *Capreolus capreolus* and *Ixodes ricinus* ticks from Norway

**DOI:** 10.1186/s13071-018-3256-z

**Published:** 2019-01-03

**Authors:** Vetle M. Stigum, Ryanne I. Jaarsma, Hein Sprong, Christer M. Rolandsen, Atle Mysterud

**Affiliations:** 1 Centre for Ecological and Evolutionary Synthesis (CEES), Department of Biosciences, University of Oslo, P.O. Box 1066 Blindern, NO-0316 Oslo, Norway; 2 Centre for Infectious Disease Control (CIb), National Institute for Public Health and the Environment (RIVM), Bilthoven, the Netherlands; 3 Norwegian Institute for Nature Research, PO Box 5685, Sluppen, NO-7485 Trondheim, Norway; 4 Evolutionary Ecology Group, Department of Biology, University of Antwerp, Universiteitsplein 1, 2610 Wilrijk, Belgium

**Keywords:** Cervids, *Ixodes ricinus*, Ticks, Tick-borne diseases, Transmission hosts, Ecotypes

## Abstract

**Background:**

The geographical expansion of the tick *Ixodes ricinus* in northern Europe is a serious concern for animal and human health. The pathogen *Anaplasma phagocytophilum* is transmitted by ticks and causes emergences of tick-borne fever (anaplasmosis) in livestock. The transmission dynamics of the different ecotypes of *A. phagocytophilum* in the ecosystems is only partly determined. Red deer and roe deer contribute to circulation of different ecotypes of *A. phagocytophilum* in continental Europe, while the role of moose for circulation of different ecotypes is not fully established but an important issue in northern Europe.

**Methods:**

We determined infection prevalence and ecotypes of *A. phagocytophilum* in moose (*n* = 111), red deer (*n* = 141), roe deer (*n* = 28) and questing ticks (*n* = 9241) in Norway.

**Results:**

As previously described, red deer was exclusively linked to circulation of ecotype I, while roe deer was exclusively linked to circulation of ecotype II. Surprisingly, we found 58% ecotype I (*n* = 19) and 42% of ecotype II (*n* = 14) in moose. Both ecotypes were found in questing ticks in areas with multiple cervid species present, while only ecotype I was found in ticks in a region with only red deer present. Hence, the geographical distribution of ecotypes in ticks followed the distribution of cervid species present in a given region and their link to ecotype I and II.

**Conclusions:**

Moose probably function as reservoirs for both ecotype I and II, indicating that the ecotypes of *A. phagocytophilum* are not entirely host-specific and have overlapping niches. The disease hazard depends also on both host abundance and the number of immature ticks fed by each host. Our study provides novel insights in the northern distribution and expansion of tick-borne fever.

## Background

Pathogens transmitted by *Ixodes ricinus* cause disease emergence in northern areas of Europe with serious implications for animal and human health [1–3]. Changes in the distribution and local abundances of the tick *Ixodes ricinus* is one of the main factors for increases in disease incidences [4], but several other processes, such as changes in vertebrate communities, also affect disease incidences [5, 6]. Fundamental knowledge of disease hazard requires understanding of the role of vertebrate hosts in the intricate links between the tick life-cycle and the enzootic cycles of the pathogens they transmit [7]. The distribution and abundance of tick-borne pathogens often depends on distribution of competent transmission hosts, not just distribution of the vector, and such insight provides another potential key to disease mitigation efforts.

*Anaplasma phagocytophilum* is the causative agent of human granulocytic anaplasmosis [8], and it also causes disease and economic losses in livestock [9]. Tick-borne fever, caused by *A. phagocytophilum*, is considered a major problem for livestock production. Anaplasmosis leads to mortality and especially lambs are at risk [10]. Infection with *A. phagocytophilum* also reduces body growth of lambs [11]. Some tick-borne pathogens, such as *Borrelia burgdorferi* (*sensu lato*) and *A. phagocytophilum*, have a high genetic diversity. Due to the high genetic diversity, *A. phagocytophilum* was formerly even split in three different species [12]. This genetic diversity has probably arisen as a response to evasion of immune defenses of phylogenetically different vertebrate host groups [13].

Elucidating the presence of different enzootic cycles for *A. phagocytophilum* is important to be able to determine which ones are pathogenic for livestock and which ones for humans, but the list of variants and transmission hosts are still incompletely understood [14, 15]. Common markers used for genotyping are *16S*-rDNA, *groEL*, *ankA* and *msp4* [14]. One classification of the *groEL* gene has identified the circulation of four ecotypes among vertebrates of Europe [15]. We define in this paper an ecotype as a cluster of genetically similar *A. phagocytophilum* isolates based on *groEL* sequences following Jahfari et al. [15]. Ecotype I based on this classification is linked to a wide range of mammals including red deer (*Cervus elaphus*), livestock and humans, whereas ecotype II is linked mainly to roe deer (*Capreolus capreolus*). Both ecotypes are most probably vectored by *I. ricinus.* Ecotype III is associated with small mammals and expected to be vectored by *Ixodes trianguliceps* [16], while ecotype IV is linked to birds and potentially vectored by *I. frontalis* [15]. In support of this scheme, the ecotype II found in roe deer was for example not the one found in livestock in France [17]. However, due to scarcity of studies, this classification in ecotypes can be considered as a working hypothesis of circulation and transmission, rather than as a definitive answer.

*Ixodes ricinus* ticks are now expanding their distribution range towards northern latitudes [2, 18]. This also involves a shift into the distribution range of another community of mammals compared to continental Europe. In the case of *A. phagocytophilum*, potential cervid transmission hosts are no longer limited to roe deer and red deer, but also include moose (*Alces alces*). Moose is by far the most important game species in terms of meat and economy both in Sweden and Norway, with an annual harvest in 2016 of, respectively, 82,097 [19] and 30,829 [20] individuals. Moose is widespread in Fennoscandia, and it is important to assess its role in the propagation of ticks and the transmission of tick-borne diseases [21, 22]. Yet, we do not know much about the potential role of moose as a transmission host of *A. phagocytophilum*. Seroprevalence for *A. phagocytophilum* of moose in Norway [22, 23], and infection levels of moose in both Norway [24] and Sweden [21] was reported to be high. However, the ecotype of *A. phagocytophilum* has only been determined in two moose with no firm conclusion, as one type I and one type II was found (data from [21] reported in [15]). Further, the geographical distribution of red deer is quite rapidly expanding in both Norway and Sweden. This may introduce the assumed pathogenic variant (ecotype I) to new areas, currently with low incidence of anaplasmosis [3, 25]. Verification of presence of a pathogenic variant in cervids is therefore important.

We here report infection prevalence and determine ecotypes of *A. phagocytophilum* in moose (*n* = 111), red deer (*n* = 141), roe deer (*n* = 28) as well as in 9241 questing *Ixodes ricinus* ticks (nymphs, male and female adults) from three regions in Norway.

## Methods

### Study areas

**Fig. 1:**
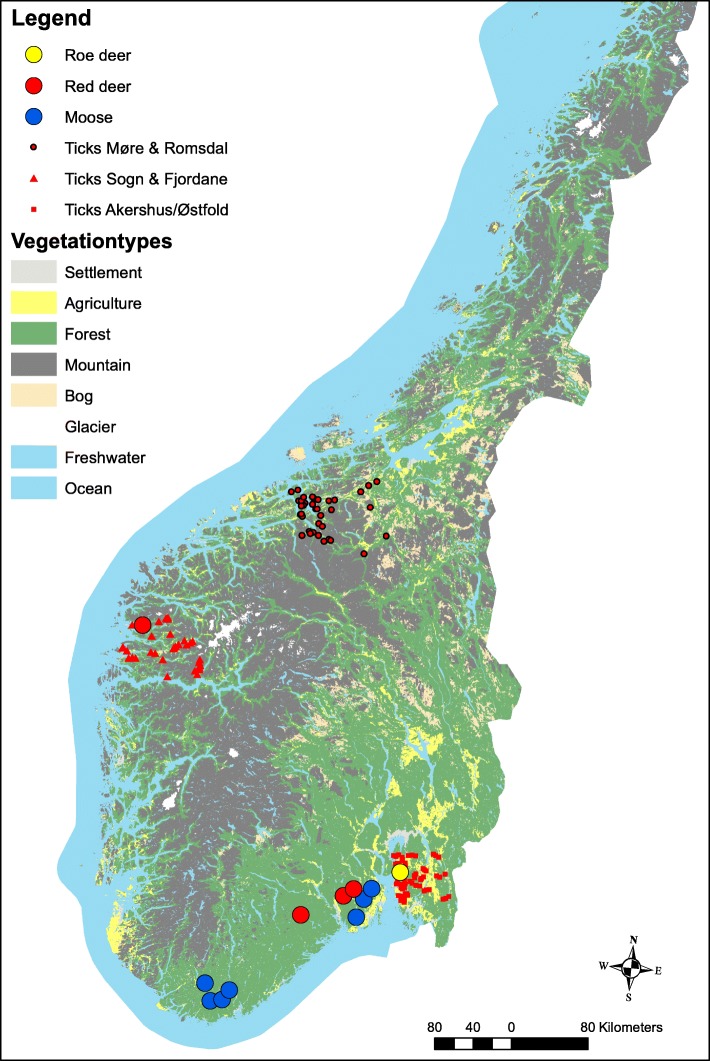
A map of the different study sites in Norway. Data on questing ticks comes from long-term monitoring in fixed transects, while samples of tissue from cervids originate from harvested animals at the scale of municipality

Samples come from different sites in the southern part of Norway (Fig. 1). Roe deer came from around Vestby municipality, Akershus county; moose came from Siljan municipality in Telemark county, Vennesla, Songdalen, Audnedal and Marnardal municipality in Vest-Agder county, and Andebu, Lardal and Re municipality in Vestfold county; red deer came from Florø municipality, Sogn & Fjordane county, Drangedal and Skien municipality in Telemark county and Lardal in Vestfold county; questing ticks came from counties Møre & Romsdal and Sogn og Fjordane in the west and Akershus and Østfold in the east.

### Sample collection and DNA extraction

Questing ticks were sampled by aid of flagging along transects in Sogn og Fjordane, Møre og Romsdal and Akershus and Østfold counties, Norway. Further details on sampling protocols of ticks can be found elsewhere [25–27]. For ticks, as in our previous work [25, 28], DNA extraction was optimized by modifying the incubation step of Qiagen DNeasy® 96 Blood & Tissue kit as recommended [29]. We incubated ticks, 2 mm zirconium oxide beads, 40 μl of proteinase K solution and 4 μl Antifoam-A (Sigma) at 56 °C overnight followed by 5 min of bead homogenization at 30 cycles per second using a Qiagen Tissue Lyser II. The samples were then frozen. This was done to more easily prepare high number of samples that later could be extracted. Later we added the remaining 160 μl of proteinase K solution and the homogenized mixture was incubated for 1 h at 56 °C. Centrifugation was carried out before removing seals at any step in the protocol to prevent cross contamination of samples. We then transferred the mixture to the DN easy plates.

Traffic killed roe deer were collected during spring 2016. The roe deer (*n* = 28) were skinned, frozen and then transported to CEES, UiO. Red deer (*n* = 141) and moose (*n* = 111, 29 yearling males, 19 yearling females, 45 adult males and 18 adult females) exterior pinna (ears) were collected by the Norwegian Food Safety Authority as part of the national monitoring for Chronic Wasting Disease during fall hunting season 2016 in Norway, and then sent as frozen samples to CEES, UiO.

A small piece (1/8 cm^2^) from the frozen exterior pinna was collected with a clean disposable scalpel and used for the DNA extraction. DNA was extracted in the same way as for ticks (except bead milling) with Qiagen DNeasy 96 Blood & Tissue kit according to the manufactures recommendations. A total of 94 samples were extracted at a time, leaving two spaces empty for controls. The DNA was stored at -80 °C for later use.

### qPCR protocol

The extracted DNA was screened for *A. phagocytophilum* by realtime PCR (qPCR) in a duplex (with *B. burgdorferi*, not reported here) [30], following Allender et al. [29] and implemented at CEES, UiO as in previous work [28].

For *A. phagocytophilum,* the *msp2 gene* was targeted with the primers ApMSP2f (5'-ATG GAA GGT AGT GTT GGT TAT GGT ATT-3') and ApMSP2r (5'-TTG GTC TTG AAG CGC TCG TA-3') and the probe ApMSP2p-[HEX]TGG TGC CAG GGT TGA GCT TGA GAT TG[TAMRA]. Each duplex qPCR reaction was done in a total volume of 10 μl, with 1 μl of extracted DNA added to the mastermix as a template. The mastermix consisted of PCR buffer (5×) (TaqMan® polymerase, LightCycler®, Roche, Basel, Switzerland) and PCR water, 900 nM for both the ApMSP2f and ApMSP2r primers and 125 nM for the ApMSP2p probe, while for *B. burgdorferi* (*s.l.*) it was 700 nM for both primers and 175 nM for the probe. Each 96-well plate contained a negative and a positive control. A two-step program was used on the LightCycler® 96 System (Roche). It started with a pre incubation of 10 min at 95 °C, followed by 50 cycles of a two-step amplification with (i) 15 s at 95 °C and (ii) 60 s at 60 °C.

### Sequencing of *groEL* from positive samples

The DNA from the samples that came up positive from the qPCR were amplified by conventional PCR, targeting a 530 bp fragment of the *groEL* gene of *A. phagocytophilum*. The Hotstart mastermix (Qiagen, Hilden, Germany) was mixed with primers and template. We used the primers EphplgroEL-A.phago-F (5'-ATG GTA TGC AGT TTG ATC GC-3') and EphgroEL-A.phago-R (5'-TTG AGT ACA GCA ACA CCA CCG GAA-3') for PCR amplification [31].

The PCR settings were a pre incubation of 95 °C for 15 min followed by 40 cycles of (i) 30 s at 94 °C, (ii) 30 s at 57 °C, (iii) 45 s at 72 °C, and a final extension of 10 min at 72 °C. To ensure that a product from the PCR was obtained, gel electrophoreses was utilized with 8 μl of DNA on a 1.5% agarose gel. The gel was stained with SYBR™ Gold Nucleic Acid Gel Stain (Invitrogen, Waltham, MA). If the PCR was successful, shown with a clear band on the gel, the PCR product was cleaned with ExoSAP-IT™ PCR Product Cleanup Reagent (Applied Biosystems, Waltham, MA) and sent for sequencing by the firm BaseClear (Leiden, the Netherlands). The chromatographs of the sequences were visually inspected and the primers sites were trimmed in Bionumerics version 7.6 (Applied Math, Belgium). Our sequences and those of known ecotypes were aligned and cluster analysis was performed as described in Jahfari et al. [15]. The sequences from this study were deposited in the Genbank database under the accession numbers MK069678-MK069965.

### Statistical analyses

Infection prevalence is binomial and therefore analysed with mixed-effects logistic regression models in R package *lme4* [32]. We used municipality as a random intercept to account for potential dependency of observations from the same region. For moose, we had data on sex and age class (yearling/adult) for all and carcass mass (kg) for 92 individuals. Carcass mass was used as a proxy for body mass [33]. We used the Akaike information criterion to establish the most parsimonious model. For red deer, there were too few negative individuals to allow a meaningful analysis of infection pattern. The statistical modeling were done in R version 3.4.2.

We made a phylogenetic tree of the sequences to explore the link between *A. phagocytophilum groEL* sequences, host and region. The sequences were aligned and trimmed in MAFFT v.7.271. A maximum clade credibility tree was prepared with BEAST v1.8.4, and visualized in Figtree v1.4.3. When plotted in QGIS v. 2.18.7 [34], the coordinates of the 288 samples formed three clusters, which we have visualized in the geographical map that was extracted from the GADM database v. 3.4 [35]. The southern cluster includes samples around the Oslofjord inlet. While Oslofjord forms a barrier, the scarceness of sampling on the east side made us chose to combine it into one location.

## Results

*Anaplasma phagocytophilum* DNA was found in 70% of the moose, in 82% of the roe deer and in 94% of the red deer (Table 1). The best model of infection prevalence in moose included both age and sex, but not the interaction term (ΔAIC = 1.592). Infection prevalence in moose was higher in males (76%) than females (59%) (*Z* = 2.175, *df* = 1, *P* = 0.030), but infection prevalence was not related to age class (*Z* = 1.553, *df* = 1, *P* = 0.120). For the subsample with known body mass, adding (log) body mass did not improve the model with age and sex included (ΔAIC = 1.958).

**Table 1:** An overview of sample sizes from various counties of Norway, the number and proportion of samples positive for *A. phagocytophilum*, the number and proportion of successful *groEL* sequences, and the number of samples identified as Ecotype I and II

Organism	County	Total	Positives		Sequences obtained		Ecotype	
		*n*	*n*	%	*n*	%	I	II
Questing ticks								
Nymph	Sogn & Fjordane	4857	238	4.90	53	22.27	53	
Adult female	Sogn & Fjordane	624	73	11.70	29	39.73	29	
Adult male	Sogn & Fjordane	625	80	12.80	20	25.00	20	
Nymph	Møre & Romsdal	1619	82	5.06	23	28.05	22	1
Adult female	Møre & Romsdal	205	36	17.56	9	25.00	7	2
Adult male	Møre & Romsdal	239	26	10.88	5	19.23	5	
Nymph	Akershus, Østfold	872	9	1.03	1	11.11	1	
Adult female	Akershus, Østfold	91	3	3.30	0	0		
Adult male	Akershus, Østfold	109	8	7.34	1	12.50		1
Cervids								
Roe deer	Akershus	28	23	82.14	13	56.52		13
Moose	Vest-Agder	51	39	76.47	19	48.72	11	8
	Telemark, Vestfold	60	39	65.00	14	35.90	8	6
Red deer	Sogn & Fjordane	126	120	95.24	73	60.83	73	
	Telemark, Vestfold	15	12	80.00	2	16.67	2	

**Fig. 2:**
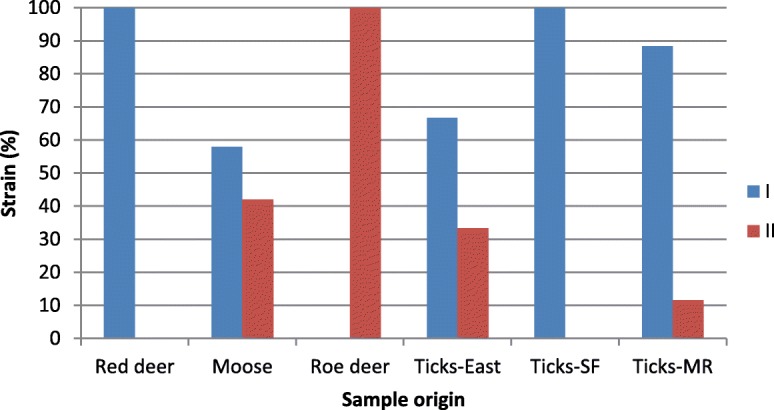
The grouping of ecotypes of *A. phagocytophilum* derived from roe deer, red deer and moose, and from questing ticks from Akershus/Østfold counties (‘east’), Sogn og Fjordane county (‘SF’) and Møre & Romsdal county (‘MR’) in Norway. Sample sizes are given in Table 1

To distinguish between ecotype I and II of *A. phagocytophilu*m, we amplified, sequenced and typed a 530 bp fragment of the *groEL* gene. We obtained successful sequences of the *A. phagocytophilum* positive samples in 42%, 57% and 57% of cases deriving from, respectively, moose, red deer and roe deer (Table 1). In moose, we found 58% ecotype I (*n* = 19) and 42% of ecotype II (*n* = 14) (Fig. 2). In red deer, all sequences grouped to ecotype I (*n* = 75). In roe deer, all sequences grouped to ecotype II (*n* = 13).

In questing ticks, we found ecotype I (*n* = 2) and ecotype II (*n* = 1) from a region with roe deer and moose (Akershus and Østfold county). Only ecotype I (*n* = 102) was found in Sogn & Fjordane county with only red deer present, and we found ecotype I (*n* = 38) and ecotype II (*n* = 5) in Møre & Romsdal county with mainly red deer and some roe deer populations (Fig. 2).

**Fig. 3:**
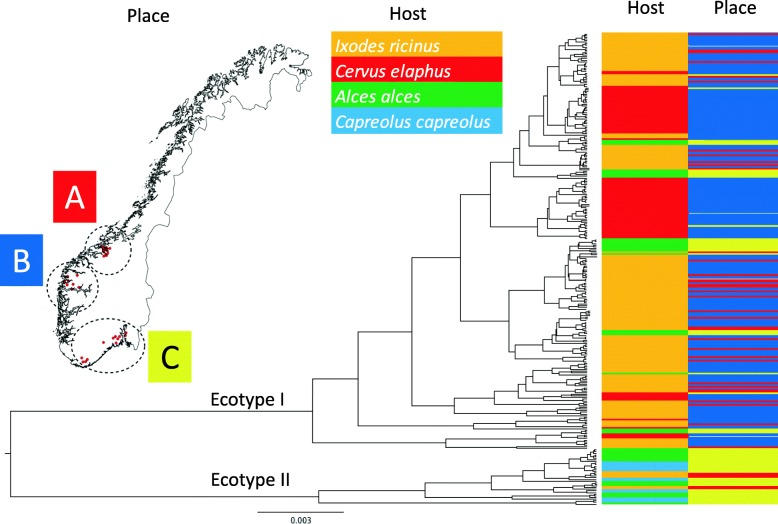
The grouping of *groEL* sequences of *A. phagocytophilum* derived from roe deer, red deer, moose and questing *I. ricinus* ticks from Norway. A, Møre & Romsdal county; B, Sogn & Fjordane county; C, placement of samples from areas in south of Norway

The phylogenetic analysis identified the two main clusters belonging to ecotypes I and II. The more fine scale genetic variation within ecotypes showed no clear geographical pattern or further association with host (Fig. 3).

## Discussion

Our study delineated ecotypes of *A. phagocytophilum* by standard molecular methods, based on the presence of DNA and bacterial *groEL* sequences originating from ear tissue of three species of cervids as well as in questing ticks coming from the northern distribution range of *I. ricinus* ticks in Europe. This study corroborates the preferential association of *A. phagocytophilum* ecotype I to red deer and ecotype II to roe deer, consistent with previous studies [15]. Importantly and surprisingly, we found evidence of high infection prevalence of both ecotype I and II in moose. Infection prevalence is not direct evidence of transmission competence. However, the high infection rates in moose and the presence of ecotype I in questing ticks in a region without red deer, but with moose present, is at least suggestive that moose may play a role in transmission of both ecotype I and II of *A. phagocytophilum.*

Presence of bacterial DNA in tissue samples is not sufficient to document the viability or infectivity of a pathogen in the host. Culturing is the gold standard for proving the viability or infectiousness of microorganisms, but this is costly, time-consuming, difficult for many tick-borne pathogens, and generally suffers from a low sensitivity [36]. PCR-array also has its limits, and there might be presence of bacteria below detectable concentrations [37]. Also, vector competence requires larval (or nymphal) ticks to be infected from their blood meal and passing viable pathogens at the nymphal (or adult female) stage to a novel host. However, since many studies are indicating *I. ricinus* as the vector and cervids as potential hosts of *A. phagocytophilum*, DNA-based detection provides useful information when aiming to elucidate the different enzootic cycles. There are, however, several other difficult issues in determining circulation and enzootic cycles of pathogens. Adaptations of bacteria to specific host groups require mutations in coding genes, but many mutations are neutral and single mutation sequence dissimilarity is not sufficient in the meaning of ecotype characterization or bacterial speciation [38]. That we found both ecotype I and II in moose, can be seen as evidence that ecotype are not entirely host-specific and have overlapping niches. The distinction between ecotype I and II are less marked than the distinction between ecotype I/II and the other two ecotypes, involving many different ruminant hosts. The *ankA* gene identifies some of the same correlation to host species as *groEL* [14]. Both methods group sequences so that roe deer is probably not the major reservoir of granulocytic anaplasmosis in humans and domestic animals, and that red deer could be the reservoir for that ecotype. A limitation of our methodology is that co-infection of ecotypes would not be discovered. The high infection prevalence of both ecotypes in moose makes it likely that mixed infections can be common. This could be addressed with other methods such as Reverse Line Blot in future work.

Obtaining sequence data from positive samples of *A. phagocytophilum* required for determination of ecotype can be challenging. We had a fairly low success (42–57%) in obtaining sequence data, and the sequencing success was good when Ct values (i.e. the number of PCR cycles before getting a positive signal) were below 30. This highlights the need for large sample sizes. There are only two previous *groEL* sequences reported from *A. phagocytophilum* isolated from moose tissue. One sequence derived from a mainland moose in Sweden was an exact match of the sequence (GQ988754) from roe deer in Austria [39] clustering to ecotype II, while the sequence derived from a moose from the Island of Öland, Sweden clustered to ecotype I [15]. There are no red deer on Öland, but still ecotype I was found there. Together with our discovery of frequent infection of ecotype I in moose, as well as in questing ticks in an area without red deer, this is indeed indicative of moose as a potential transmission host of ecotype I.

## Conclusion

Our study provides one crucial step forward towards identifying assumed transmission hosts at the northern distribution ranges, and we have provided the first results potentially linking moose to both ecotype I and II of *A. phagocytophilum*. The importance of a given host for the disease hazard also depends on their abundance and the number of ticks feeding on them [40, 41]. Red deer can generally reach higher population densities than moose, while roe deer have highly variable densities that can be very high locally [42]. We found a considerable amount of larvae attached to red deer close to the present western study site in Norway [43], and larvae were also found on roe deer in Germany [44]. Lower proportion of larval ticks has been found on moose ears in Norway when compared to red deer and roe deer [45]. We found much lower prevalence of *A. phagocytophilum* in questing ticks from the eastern study site with roe deer and moose compared to in the western study sites with red deer [25]. Therefore, we still need more quantitative data to better understand the role different cervid species play in determining disease hazard.
